# Manual Loading Distribution During Carrying Behaviors: Implications for the Evolution of the Hominin Hand

**DOI:** 10.1371/journal.pone.0163801

**Published:** 2016-10-03

**Authors:** Alastair J. M. Key

**Affiliations:** School of Anthropology and Conservation, University of Kent, Canterbury, Kent, CT2 7NR, United Kingdom; Museum für Naturkunde, GERMANY

## Abstract

The human hand is unparalleled amongst primates in its ability to manipulate objects forcefully and dexterously. Previous research has predominantly sought to explain the evolution of these capabilities through an adaptive relationship between more modern human-like anatomical features in the upper limb and increased stone tool production and use proficiency. To date, however, we know little about the influence that other manipulatively demanding behaviors may have had upon the evolution of the human hand. The present study addresses one aspect of this deficiency by examining the recruitment of the distal phalanges during a range of manual transportation (i.e., carrying) events related to hominin behavioral repertoires during the Plio-Pleistocene. Specifically, forces on the volar pad of each digit are recorded during the transportation of stones and wooden branches that vary in weight and size. Results indicate that in most instances, the index and middle fingers are recruited to a significantly greater extent than the other three digits during carrying events. Relative force differences between digits were, however, dependent upon the size and weight of the object transported. Carrying behaviors therefore appear unlikely to have contributed to the evolution of the robust thumb anatomy observed in the human hand. Rather, results suggest that the manual transportation of objects may plausibly have influenced the evolution of the human gripping capabilities and the 3^rd^ metacarpal styloid process.

## Introduction

The ability of modern humans to manipulate, modify, and utilize aspects of our physical environment is unparalleled amongst primates. This is due to a suite of musculoskeletal adaptations that allow modern humans to effectively undertake fine manipulative behaviors, forceful precision grips, and powerful ‘squeeze’ grips, all of which are more limited in other primate species [1–5]. The advent of modern human-like hand anatomy therefore represents a key event in human evolutionary history. In recent years, discussion on this matter has been aided by a suite of fossil discoveries [6–10], from which, an increasingly detailed picture of hominin hand anatomy is starting to emerge [2, 11]. In addition, new analytical techniques and experimental procedures are broadening our understanding of how the behavior of early human populations may have been influencing the anatomy of, and selective pressure acting upon, hominin hands [12–19].

The analysis and interpretation of fossil hominin hand remains has regularly referenced the emergence and use of stone tool technology. From Napier’s [20] pioneering analyses of the OH7 *Homo habilis* fossils through to the recent description of the *Homo naledi* hand remains [9] reference has frequently been made to a hominin’s ability to make and use stone tools (e.g., [6, 7, 21–24]). This link is because the Palaeolithic record identifies a series of known behaviors that directly (and heavily) recruit the hand (e.g. [25]), and as such, represent one of the few pieces of behavioral evidence against which fossil anatomy can be compared and evolutionary hypotheses can be constructed. Recent archaeological discoveries pushing the first suggested occurrence of intentionally flaked stone technology back to 3.3 MYA [26] therefore have implications for our understanding of the evolution of hominin manipulative capabilities. Certainly, at least one hominin species at 3.3 MYA displayed anatomical features in the hand that not only facilitated bipolar and passive hammer reduction techniques (see: [26, 27]) but were also capable of manipulating and applying the resultant flake technology during cutting behaviors [1, 28, 29]. Suggestions of human-like manipulative capabilities (and hand anatomy) in australopithecine species prior to ~3 MYA are then supported by these contemporaneous archaeological discoveries.

Fossil analyses suggesting the presence of modern human-like anatomical features in the hominin hand prior to the advent of (or in absence of) stone tool production/use, however, stress the evolutionary influence that other manipulative behaviors may have had [30–34]. Evolutionary hypotheses regarding the human hand are, however, focused upon the use and production of stone tools [1, 2, 11] (although see also: [35–37]). This is understandable given the role of stone tools within hominin behavioral repertoires and (subsequent to ~2.6 MYA) their relative prevalence in the archaeological record [38–43]. Experiments examining the human hand/upper limb during the use and production of stone tools have, for example, shown that:

- Selective pressures may plausibly have been acting upon the hand of early hominins as a result of their influence over stone tool performance characteristics [15, 17].
- Modern human motor processes and upper limb biomechanical capabilities facilitate the effective production of stone tools [12, 13, 44, 45].
- Muscular and skeletal anatomy serving the thumb, index and fifth digit are heavily recruited during stone tool use and production behaviors [12, 14, 18, 35, 46].
- Stone tool use and production necessitates a number of grips that are unique to the manipulative capabilities of humans in terms of their ability to be forcefully and/or effectively undertaken [1, 47].

While these investigations provide valuable data that are relevant to the evolution of the hand subsequent to the onset of the earliest flaked stone technology (currently *circa* 3.3 MYA), they are limited to a set of behaviors that would only have formed part of the total manipulative repertoire that influenced the evolution of the hominin hand. Indeed, there is a near absence of experimental data relating to the manipulative demands likely experienced by early humans during other manual activities.

Chimpanzees (*Pan troglodytes*) are known for their tool use capabilities. This includes relatively complex tool production processes, forceful thrusting of branches, heavy-duty percussive processes, and the relatively fine application of termite fishing sticks (e.g. [48–53]). These behaviors are, in varying ways, manipulatively demanding. Recent findings have also shown that wild chimpanzees employ precision grips with a degree of force during a range of non-tool use related tasks [37, 54]. Marzke et al. [37], for instance, observed chimpanzees heavily recruiting their thumb and index finger when stabilizing food items against the pull of the teeth during feeding. Similar observations have previously been made in relation to other great ape species [31]. Parsimony therefore suggests that early humans were likely undertaking similar behaviors with their hands. Relatedly, chimpanzees are known to carry wooden/stone hammers and anvils over considerable distances (on occasion >500m) in preparation for percussive food processing [55–57]. Moreover, these materials can be of a substantial weight (e.g. >5kg), occasionally weighing up to 15kg [56, 58]. It is therefore reasonable to suggest that early hominins were also likely to have been transporting objects of considerable weight for prolonged periods [59]. This statement is supported by archaeological evidence from the Oldowan identifying raw materials being transported up to 13km [60–63]. Notably, the distances reported by these archaeological studies are far greater than those observed in studies of extant primates, demonstrating that early hominins were exceptional relative to extant non-human primate species in the extent to which they transported objects [59, 64]. Poor preservation of organic materials impedes discussion of non-lithic related manipulative behaviors by fossil hominins. Nonetheless, there is evidence of early hominin interactions with plant and animal (bone) materials, including their transportation and use as tools [38, 39, 42, 65–71]. In sum, not only were manipulatively demanding behaviors not related to the use or production of stone tools likely to have frequently been undertaken by early hominins, but there is evidence to suggest that early hominins manually transported objects over distances far in excess of those reported for extant primates. To date, however, very little is known about the influence that these activities may have had upon the evolution of the human hand, or how they may influence the interpretation of fossil hand remains [31].

Given these foregoing factors, it would be valuable if data were available that detailed the musculoskeletal stresses enacted upon the hand by Plio-Pleistocene manipulative behaviors that do not relate to stone tool production or use. Indeed, not only would this allow more accurate functional interpretations of fossil hominin hand anatomy, but it would facilitate increased understanding of the framework within which modern human-like anatomical features evolved. Here, I examine the stresses experienced by the thumb and fingers during the transportation of raw materials associated with hominin behavioral repertoires during the Plio-Pleistocene. Specifically, force sensitive sensors are attached to the distal pads of the thumb and fingers of 32 individuals while they carry stones and wooden branches of varying weights and sizes. Relative force distributions between the five digits, and their relationship with an object’s size and weight, are used to examine the influence that carrying behaviors may have had upon the evolution of human hand anatomy.

## Materials and Methods

### Participants

32 participants were recruited from the student population at the University of Kent, Canterbury (UK). Participant ages ranged between 18–43 years (mean = 22) and there was a female to male ratio of 1:0.7. Prior to undertaking the experiment five biometric variables were collected from the hands of each individual (Table 1; S1 File). Shapiro-Wilk tests confirmed that participant biometric conditions were derived from a naturally normally distributed population (*P* = .797 - .090). All individuals were informed of the experimental protocol prior to their participation but specific research goals were not disclosed. Informed consent was obtained in writing prior to participation and ethical approval was provided by the School of Anthropology and Conservation (University of Kent) Ethics Committee.

**Table 1:** Descriptive biometric data for the 32 participants who undertook the experiment.

	Mean	Range	Standard Deviation
Hand Length (mm)	177	149–200	11.7
1^st^ to 2^nd^ digit Ratio (1^st^/2^nd^)	0.868	0.057–1.029	0.166
Grip Strength (kg)	37.9	20–67	11.3
Tip-to-Tip Pinch Strength (kg)	5.0	2.2–12	1.8
Pad-to-Side Pinch Strength (kg)	6.9	3.6–16	2.2

### Transported Materials

Chimpanzees are known to transport stone and wooden objects that vary highly in size and weight. Typically this is prior to, or during, tool-use related behaviors [53, 59]. While archaeological evidence can only confirm the transportation of stone and bone materials by Lower Palaeolithic hominins (e.g. [62, 63, 68, 70]), it is reasonable to conclude that wooden objects would similarly have been transported at this early stage in human evolution. Hence, in order to replicate manipulative demands likely experienced by Plio-Pleistocene hominins during carrying behaviors, a selection of stones and wooden branches were required to be transported by each participant.

Three distinct classifications of ‘stone objects’ were selected on the assumption that they may elicit differing manipulative force requirements. Hence, when combined with the ‘wooden branches’, four broad classifications of objects were required to be transported by participants. These were:

1. Spherical stones that vary in terms of their weight and circumference/diameter.
2. Flat stones (long and wide relative to their depth) that vary in terms of their weight and maximum dimensions.
3. Stones of weight enough to require two hands during transportation.
4. Cylindrical wooden objects that vary in their circumference and diameter but are identical in terms of their weight.

**Fig 1:**
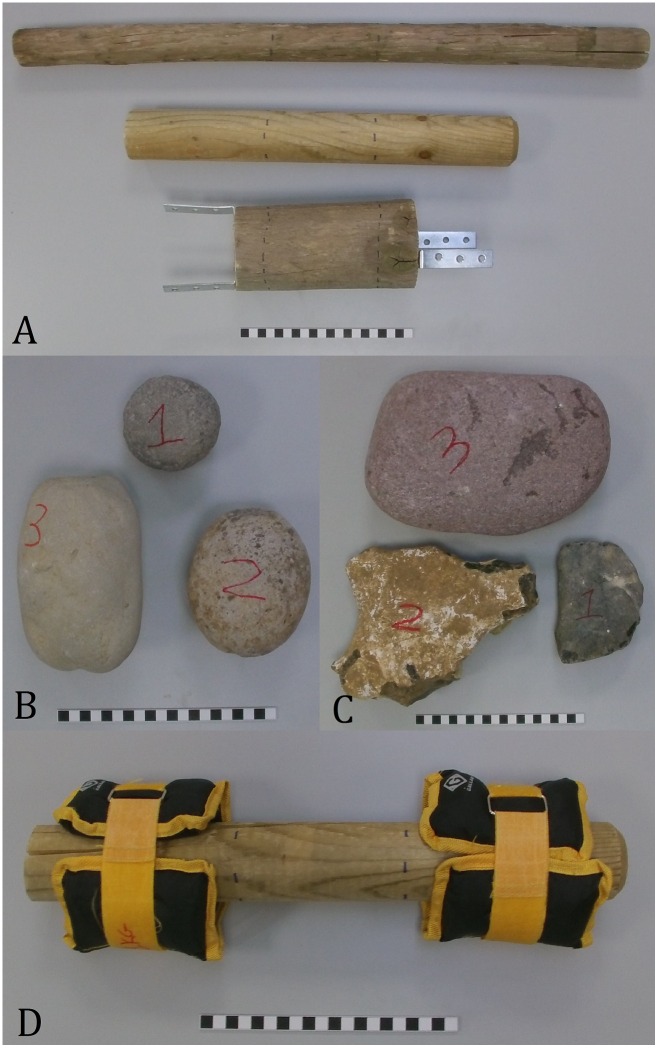
The three wooden posts (A), three round stones (B), two flat stones (C1, C2) and large two handed stone (C3) carried by participants during this experiment. Image (D) demonstrates how the wrist weights were attached to the three wooden posts. In all instances there was a 12cm gap between the weights (identified by a dotted line). Note that the scale bar is 20cm in each image.

There were three spherical stones, two flat stones, and one stone weighing enough to require two hands during transportation (Fig 1). All participants were required to carry all of the stone objects. Descriptive data for the stone objects can be seen in Table 2. Due to the shape and surface variability of branches, the three cylindrical wooden objects utilized were industrially produced fence posts of varying diameter. The wooden posts weighed 600g in order to allow an examination of object size whilst controlling for weight variability. This resulted in the length of each wooden cylinder decreasing as circumferences/diameters increased. It was, therefore, necessary to attach additional loads to the posts in order to examine any influence that object weight may have. ‘Wrist weights’ were secured to the ends of each post to achieve this (Fig 1). In total six identical weight categories were examined for each wooden object. Weights increased in increments of 1kg, starting with the three wooden posts without additional loads (i.e. 600g), before increasing to 1.6kg, 2.6kg, 3.6kg, 4.6kg, and 5.6kg. Hence, not only were participants required to carry three wooden cylindrical objects of varying circumferences/diameters, but they were required to do so under six different weight conditions (in total, individuals carried wooden objects on 18 separate occasions) (Fig 1). This distribution of weight was chosen as an upper threshold of 5.6kg covers the majority of hammers selected and transported by chimpanzees during nut-cracking processes and the vast majority of stone cores/hammerstones/tools utilized by early hominins [56, 72, 73]. Descriptive data for the posts can be seen in Table 2. In total, participants carried 24 objects.

**Table 2:** Descriptive data for the objects transported by participants. ‘Length’ was recorded as the maximum distance parallel to the top surface of an object (as seen in Fig 1). ‘Width’ was recorded as the maximum distance perpendicular to ‘Length’ and parallel to the top surface of an object. ‘Depth’ was recorded as the maximum distance perpendicular to both ‘Length’ and ‘Width’.

	Round Stones			Flat Stones		Large Stone	Posts		
	1	2	3	1	2	1	1	2	3
**Weight (kg)**	0.67	1.65	2.5	0.94	3.2	10.4	0.6	0.6	0.6
**Diameter (cm)**	-	-	-	-	-	-	4.5	5.8	9.5
**Length (cm)**	8.1	12.0	16.9	14.0	25.0	28.0	74	44	20
**Width (cm)**	7.8	9.7	10.5	10.5	18.5	18.5	-	-	-
**Depth (cm)**	6.2	8.5	8.2	3.9	5.1	9.8	-	-	-

### Carrying Procedure

**Fig 2:**
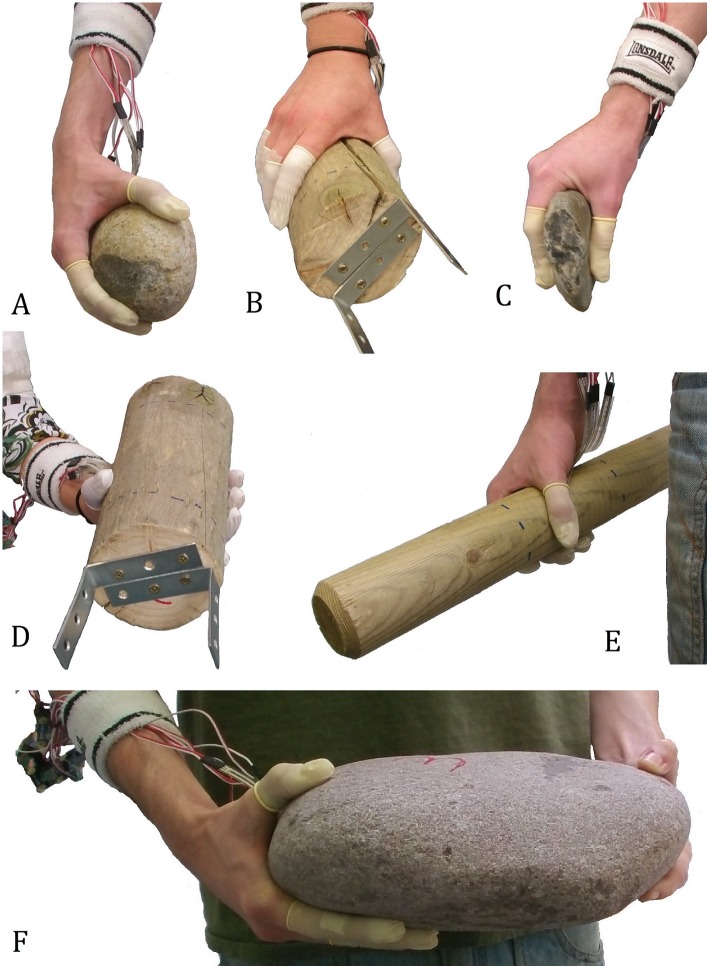
Examples of grips frequently employed by participants during the carrying events. Figs A, C and F depict the transportation of round stone two, flat stone one, and the large stone (respectively). Figs B and D both depict post three while Fig E shows post two. Further images identifying the grips employed can be seen within S1 Fig.

To control for the potential effect of fatigue the order in which the 24 objects were carried was independently randomized for each participant. Participants were required to carry each of the 24 objects along a pre-set 14 meter course. After completing the course once, participants were required to place the object down on a table before picking it up again and completing it a second time. This gave participants the chance to readjust the grip applied to the object and meant that in total, each object was carried over 28 meters. Grip choice will undoubtedly influence the distribution of force across the five digits; hence, any natural variation that participants may create in this regard was factored into analyses (i.e. force records were the product of both carrying events; see below). All but one of the 24 carrying events was required to be undertaken solely with dominant hand (Fig 2). The exception was the transportation of the large stone which required the use of both hands (Fig 2). Participants were instructed that they were allowed to grip the objects in whatever manner they preferred and all were asked to walk at a ‘comfortable’ pace.

**Fig 3:**
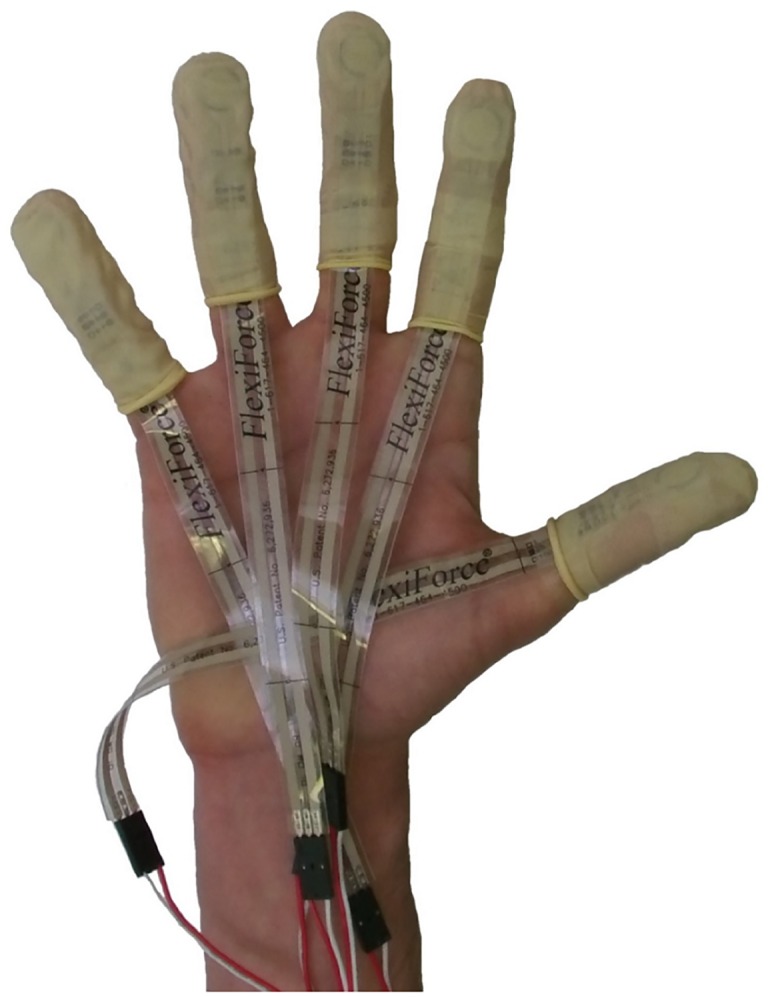
The Tekscan Flexiforce^®^ force sensors and where they were attached on the hand. The positioning of the circular (9.5mm diameter) pressure sensitive area upon the volar pad of each digit can be seen through the finger cots.

**Fig 4:**
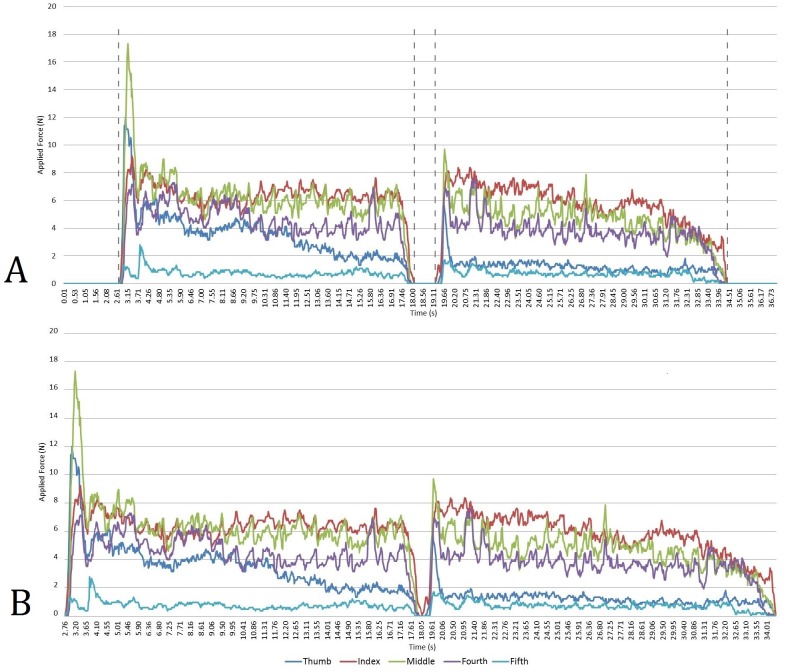
Data output during a carrying event. Image (A) indicates the two portions of the data stream that directly refer to when an object was being carried. These two portions were then merged (B) so that a mean and maximum value for each digit could be recorded across both segments of force output.

Force data were recorded using Tekscan Flexiforce^®^ force sensors (Fig 3). These are thin film sensors that that provide highly accurate manipulative force sensing capabilities and have been shown to be superior to a number of other products on the market during the measurement of force in gripping tasks [74]. Subsequent to their calibration (which allows resistance outputs to be converted into force records [Newtons]), each sensor was able to measure static normal forces acting upon its active sensing area (the 9.5 mm diameter of this area is treated as a single contact point; Fig 3). As such, force records were the product of the loads exerted and resisted by the distal phalanges at the point of contact with the transported objects. Each sensor was connected to a Phidgets^™^ Flexiforce Adapter running through a Phidgets^™^ Interface Board and a modified version of the associated software. This allowed any force data to be recorded directly onto an Excel spreadsheet. Five sensors were attached to the dominant hand of each participant. Each digit had a sensor attached to it, with the sensitive area of the sensor being secured to the volar pad (Fig 3). Hence, not only was force recorded from each of the five digits on the dominant hand, but it was recorded from the point on the distal phalanx thought to have evolved to withstand manipulative loading [75]. Medical tape and finger cots were used to keep the sensors in place during the experiment (Fig 3). As demonstrated by previous research [14, 18], if care is taken to avoid placing tape upon sensor elements this method of attachment has no impact upon force records (as demonstrated in Fig 4A).

### Data Analysis

Data were recorded at a rate of 40 Hz; this resulted in thousands of data points for every transportation event undertaken. The two portions of data that specifically referred to when an object was being carried were identified and combined (Fig 4). Mean and maximum values were then recorded for each digit across these two combined course repetitions.

Statistical analyses were undertaken to examine two main research objectives. The first set of analyses were designed to establish whether there were any differences in force between individual digits during the manual transportation (carrying) of stones and wooden branches. This would facilitate discussion on potential selective pressure experienced by aspects of the hand as a result of carrying behaviors and any traces that these behaviors may leave in the hominin fossil record. Comparisons were undertaken at a broad level, with data from the 24 objects being combined relative to their categorization as round stones (x3), flat stones (x2), very large stones (x1), or wooden posts (x18) (i.e. n = 96, 64, 32, and 576 for each digit within each object type, respectively). This was repeated for both the mean and maximum values recorded during transportation events. In most cases data violated assumptions of normality, however, due to the more limited statistical strength of non-parametric tests, the increased likelihood of Type 1 Error from their repeated use, and the additional data manipulation required by transforming data to get a normal distribution, the statistical examination of any differences between individual digits was undertaken through the randomization procedure outlined by Wood [76].

**Fig 5:**
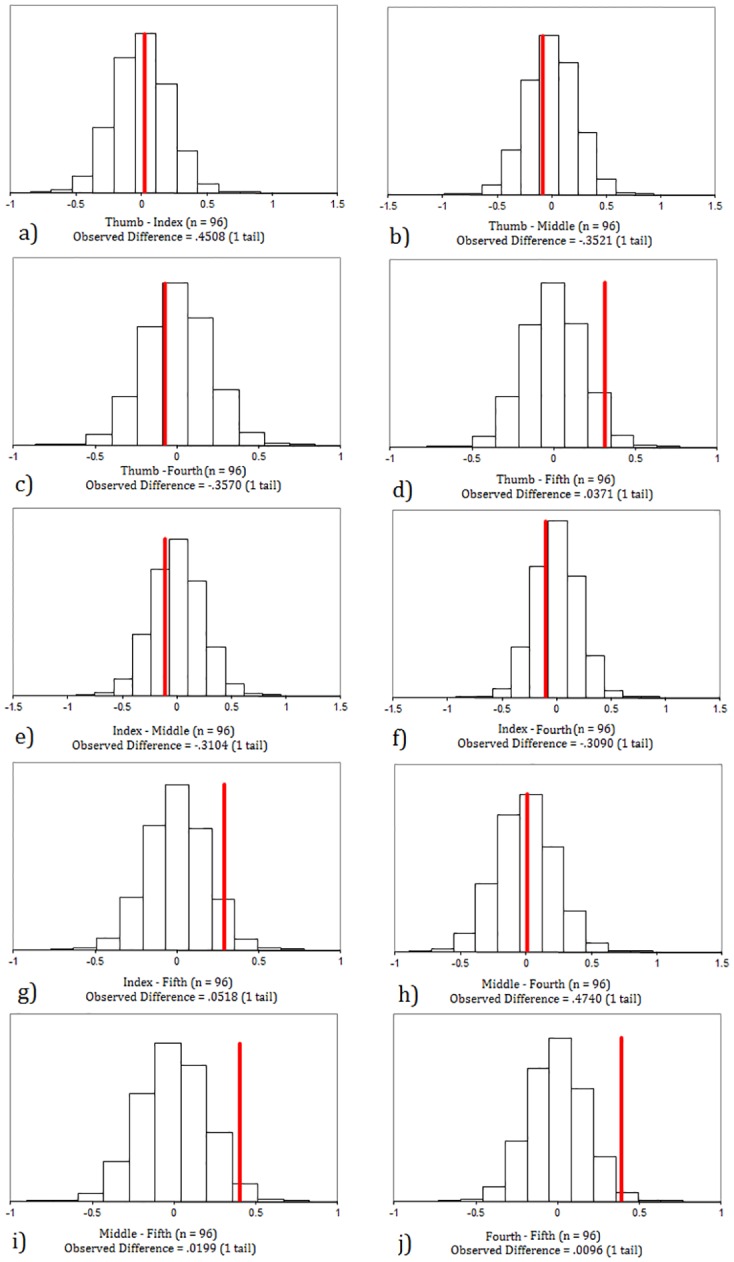
Distribution of possible differences between the mean force exerted by each digit during the transportation of the round stones (n = 96) after 100,000 resamples without replacement in each case. If this line lies in the tail end of the distribution then the difference is unlikely to have occurred by chance. The ‘observed difference’ value is expressed as a proportion of cases more extreme than the observed difference value and is analogous to a significant ‘*P’* value.

In brief, data were randomly resampled without replacement to generate a distribution of possible mean force differences between digits. These were compared to the observed differences between the digits in a manner analogous to a *P* value. The further the observed difference between the means deviated from the mean of the generated distribution, the less likely it would have occurred by chance (see: Fig 5). Computational power prevented complete enumeration of possible permutations, however each difference was resampled and generated 100,000 times allowing for a robust level of significance. This was independently repeated for both the mean and maximum force data recorded from participants.

The second objective of this investigation was to examine the influence that variation in object size and weight may have upon the forces acting upon digits during raw material transportation behaviors. Data were collected from the transportation of wooden posts so that the influence exerted by each of these variables could be examined independently. To investigate the influence that the size (i.e. diameter) of a post had upon the forces experienced by individual digits, data from the six weight categories for a given post size were combined (e.g. all weight variations for post 1 [n = 192 for each digit]). Similarly, to examine the influence that weight variation may have upon manipulative force, data from the three post sizes for any given weight category were combined (e.g. all post sizes weighted at 2.6 kg [n = 96 for each digit]). Analyses were then undertaken to compare the relative forces experienced by a digit during the transportation of one post size or weight relative to another. For example, the force acting upon the thumb during the carrying of Post 1 was compared to that when carrying Post 2 and Post 3 (n = 96 in all instances). These comparisons were achieved via the randomization procedure outlined above. Analysis of object size and weight was only undertaken for the mean force values recorded from participants.

## Results

Descriptive statistics for the force acting upon individual digits during the manual transportation of the three stone classifications and the wooden posts are detailed in Tables 3 and 4. The latter are defined relative to all 18 post transportation events being combined, the three size categories, and the six weight categories.

**Table 3:** Descriptive force (N) data for each digit during the transportation of all posts when combined, the three differing post sizes, and the six varying post weights.

			All Posts Combined (n = 576)	Post Size (n = 192)			Post Weight (kg) (n = 96)					
				1	2	3	0.6	1.6	2.6	3.6	4.6	5.6
**Thumb (N)**	**Mean**	Mean	1.3	0.8	1.0	2.2	0.9	1.5	1.2	1.2	1.8	1.5
		S.D.	1.6	1.3	1.0	2.1	1.5	1.6	1.8	1.3	1.9	1.5
	**Maximum**	Mean	8.0	3.1	3.5	8.1	5.2	5.2	4.6	4.0	5.5	5.0
		S.D.	4.8	3.4	3.0	5.8	5.2	5.0	4.6	3.9	5.2	4.7
**Index (N)**	**Mean**	Mean	2.3	1.9	2.1	2.7	0.8	1.8	1.9	2.5	3.8	2.8
		S.D.	2.3	2.0	2.0	2.7	0.9	1.6	2.1	2.1	3.1	2.0
	**Maximum**	Mean	7.7	5.7	5.8	7.8	2.8	5.9	5.6	7.0	9.0	8.1
		S.D.	5.1	4.5	4.5	6.0	2.9	3.9	5.0	5.1	6.0	5.1
**Middle (N)**	**Mean**	Mean	2.3	1.3	1.6	3.9	1.1	1.8	2.1	2.9	3.2	2.6
		S.D.	2.7	1.3	1.6	3.6	1.6	2.1	2.6	2.9	3.2	2.8
	**Maximum**	Mean	12.0	4.9	5.3	12.1	6.1	6.9	6.7	8.3	8.9	7.7
		S.D.	6.6	4.3	4.3	7.8	5.9	6.1	6.0	6.7	6.9	7.4
**Fourth (N)**	**Mean**	Mean	1.45	1.0	1.1	2.4	0.7	1.0	1.5	1.8	1.9	1.8
		S.D.	1.8	1.1	1.2	2.5	1.0	1.2	1.9	2.2	2.2	1.9
	**Maximum**	Mean	7.6	4.0	4.0	7.7	4.6	4.0	5.0	5.7	6.1	6.0
		S.D.	4.5	3.3	3.4	5.5	4.5	3.5	4.4	4.6	5.0	4.7
**Fifth (N)**	**Mean**	Mean	1.0	1.0	0.9	1.1	0.5	0.5	1.0	1.4	1.1	1.5
		S.D.	1.3	1.1	1.2	1.4	0.7	0.9	1.2	1.6	1.2	1.4
	**Maximum**	Mean	4.4	4.8	4.1	4.4	2.9	2.9	4.6	5.6	5.0	5.5
		S.D.	3.8	3.8	3.8	3.6	2.6	2.9	4.2	4.2	3.6	3.8

**Table 4:** Descriptive force (N) data for each digit during the transportation of the round, flat and large stones.

			Round Stones				Flat Stones			Large Stone
			1(n = 32)	2(n = 32)	3(n = 32)	Combined(n = 96)	1(n = 32)	2(n = 32)	Combined(n = 64)	1(n = 32)
**Thumb (N)**	Mean	Mean	0.7	1.6	1.2	1.2	0.7	1.6	1.2	1.2
		S.D.	0.9	1.9	1.1	1.4	0.8	2.1	1.6	1.5
	Maximum	Mean	3.6	8.8	7.2	6.6	4.7	5.6	5.2	3.6
		S.D.	2.8	6.6	5.6	5.6	3.1	5.2	4.3	3.1
**Index (N)**	Mean	Mean	0.7	1.1	1.7	1.2	0.5	2.1	1.3	2.1
		S.D.	0.8	1.2	1.9	1.4	0.5	1.8	1.5	2.2
	Maximum	Mean	2.4	4.0	5.5	4.0	3.2	7.0	5.1	9.8
		S.D.	2.8	3.1	4.7	3.8	3.0	5.2	4.6	6.0
**Middle (N)**	Mean	Mean	0.6	1.2	2.0	1.3	0.9	2.7	1.8	2.1
		S.D.	0.8	2.9	1.7	1.7	0.8	2.0	1.8	2.3
	Maximum	Mean	2.2	5.5	11.1	6.3	6.0	7.7	6.8	12.5
		S.D.	2.1	4.1	9.0	6.6	4.4	5.3	4.9	9.9
**Fourth (N)**	Mean	Mean	0.8	1.3	1.7	1.3	0.6	1.5	1.1	1.3
		S.D.	0.9	1.6	1.2	1.3	0.6	1.6	1.3	1.2
	Maximum	Mean	3.5	8.9	9.9	7.4	5.8	4.2	5.0	9.6
		S.D.	3.3	8.0	6.7	6.9	6.2	3.6	5.1	7.6
**Fifth (N)**	Mean	Mean	0.4	0.9	1.3	0.9	0.4	0.9	0.7	1.1
		S.D.	0.8	1.0	1.0	1.0	0.4	1.0	0.8	1.2
	Maximum	Mean	2.8	5.9	8.1	5.6	4.3	3.5	3.9	8.3
		S.D.	3.2	5.5	5.0	5.1	4.7	2.8	3.9	6.2

### Force Distribution between Digits

**Fig 6:**
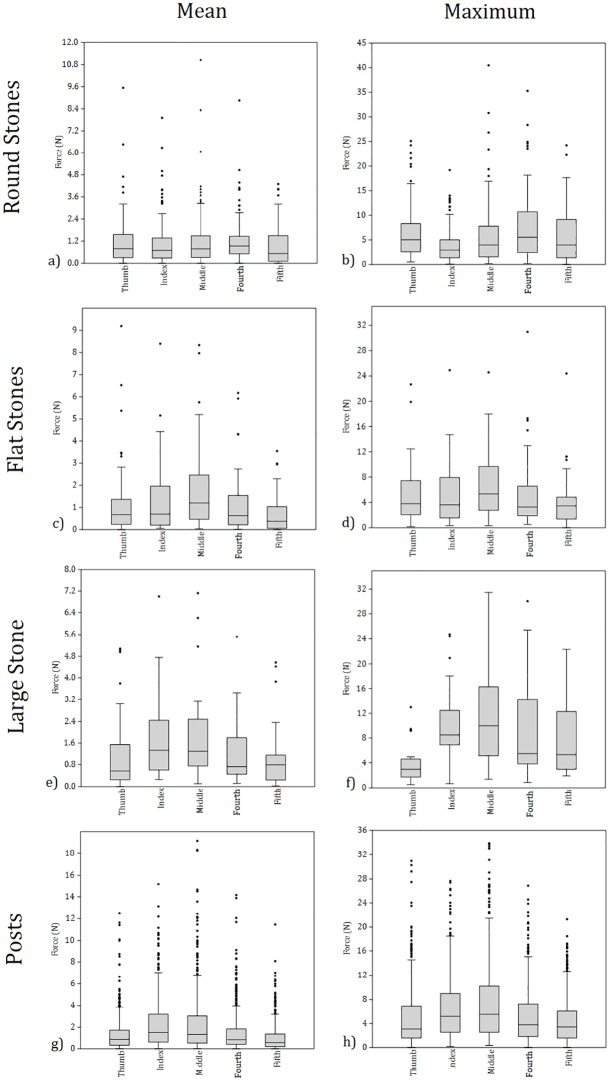
Boxplots displaying the mean and maximum force (N) recorded by individual digits during the transportation of the round (n = 96), flat (n = 64) and large (n = 32) stone classifications. Combined force values for the 18 weight and size combinations during post transportation events are also displayed (n = 576).

The first set of analyses aimed to determine whether there were statistically significant differences in the forces acting upon individual digits during the manual transportation (carrying) of stones and wooden branches. Comparisons were independently performed for both the mean and maximum force values recorded from transportation events. Results identify that significant differences do exist between digits, however, this varies dependent upon the object being carried (Table 5; Fig 6). During the transportation of the round stones, significant differences in mean force are only found when the thumb, middle finger, and fourth finger are compared against the fifth finger (Table 5; Figs 5 and 6). In all instances, the fifth finger recorded significantly lower force. Regarding maximum forces, in all instances the index finger recorded significantly lower maximal force than the other four digits (Table 5; Fig 6). In addition to this, the fourth finger recorded significantly more force than the fifth digit. All other comparisons returned non-significant results (Table 5).

**Table 5:** Relative force differences between digits during the transportation of the round stones (n = 96), the two flat stones (n = 64), the large stone transported with two hands (n = 32), and all 18 combinations of post sizes and weights (n = 576). Identified here are the proportion of resample cases more extreme than the difference recorded after 100,000 resamples without replacement (one tail). These values are analogous to a significant ‘*P*’ value, with significant differences (where cases more extreme than the observed difference are under 5% of the distribution) being highlight in bold. Positive values indicate the digit detailed in the vertical column to have the greater of the comparative forces, while a negative value indicates the digit in the horizontal row to be greater. Results are displayed for both mean and maximum force values.

		Mean Force					Maximum Force				
**Round Stones**	(n = 96)	Thumb	Index	Middle	Fourth	Fifth	Thumb	Index	Middle	Fourth	Fifth
	Index	.4508					**.0001**				
	Middle	-.3521	-.3104				.3885	**-.0018**			
	Fourth	-.3570	-.3090	.4740			-.1718	**-.0001**	-.1295		
	Fifth	**.0371**	.0518	**.0199**	**.0096**		.1081	**-.0068**	.2183	**.0184**	
**Flat Stones**	(n = 64)	Thumb	Index	Middle	Fourth	Fifth	Thumb	Index	Middle	Fourth	Fifth
	Index	-.3596					.4631				
	Middle	**-.0252**	**-.0477**				**-.0208**	**-.0193**			
	Fourth	.3419	.2015	**.0056**			.4248	.4631	**.0194**		
	Fifth	**.0101**	**.0017**	**.0001**	**.0152**		**.0390**	.0552	**.0001**	.0837	
**Large Stone**	(n = 32)	Thumb	Index	Middle	Fourth	Fifth	Thumb	Index	Middle	Fourth	Fifth
	Index	**-.0452**					**-.0001**				
	Middle	**-.0357**	-.4458				**-.0001**	-.0998			
	Fourth	-.4918	**.0344**	**.0274**			**-.0001**	.4427	.0961		
	Fifth	.3019	**.0113**	**.0089**	.2648		**-.0002**	.1593	**.0213**	.2308	
**Posts**	(n = 576)	Thumb	Index	Middle	Fourth	Fifth	Thumb	Index	Middle	Fourth	Fifth
	Index	**-.0001**					**-.0001**				
	Middle	**-.0001**	-.4868				**-.0001**	.1710			
	Fourth	-.1550	**.0001**	**.0001**			-.0850	**.0001**	**.0001**		
	Fifth	.0703	**.0001**	**.0001**	.1495		-.0595	**.0001**	**.0098**	**.0012**	

During the transportation of the two flat stones the greatest force was recorded by the middle finger, with this being to a significant extent relative to the four other digits for both mean and maximum force data (Table 5; Fig 6). The fifth finger recorded significantly less force than the other four digits when mean values were compared, and significantly less force than the thumb and middle finger when maximum forces were compared. All other differences between digits were non-significant (Table 5). During the transportation of the large stone that was sufficiently heavy to require two hands, the index and middle fingers recorded significantly greater mean forces relative to the thumb and the fourth and fifth fingers (Table 5; Fig 6). The thumb recorded significantly lower maximal forces in all instances (Table 5; Fig 6). The only other significant difference between maximal forces was identified between the middle and fifth fingers, where the former was recruited to a greater extent. Force differences between digits during the transportation of the wooden posts are similar between the mean and maximum force data. In both instances, the index and middle finger recorded significantly more force than the thumb and the fourth and fifth fingers (Table 5; Fig 6). All but one other comparison (fourth-fifth, maximal force) returned non-significant differences. Notably, these relationships are relatively constant across all three post sizes (S1 Table).

### Force Relative to Object Size and Weight

**Fig 7:**
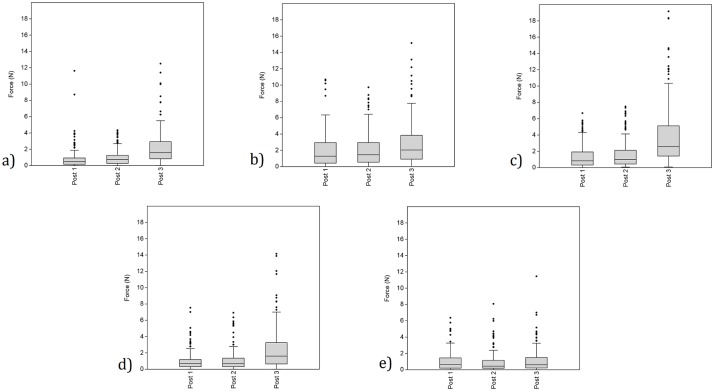
Boxplots identifying the mean force (N) recorded by individual digits during the transportation of the three post sizes (all post weights combined, n = 192). Images A, B, C, D, and E identify the thumb, index, middle, fourth and fifth fingers (respectively).

The second set of analyses examined what influence increases to the size or weight of an object may have upon the force recorded by each digit during carrying behaviors. Object size was investigated first, with the mean forces recorded by digits during the transportation of the three wooden posts being compared. Comparisons between Post 1 and Post 2 only demonstrated a significant difference for one digit. In this instance, the middle finger recorded significantly greater force during the transportation of Post 2 relative to Post 1 (Table 6; Fig 7). Both of the force comparisons with Post 3 identified significant differences for digits 1–4. That is, relative to Posts 1 and 2, the transportation of Post 3 resulted in significantly greater amounts of force being recorded by the thumb, index, middle, and fourth fingers (Table 6; Fig 7). Contrary to the other four digits, no significant differences were identified for any of the post size comparisons for the fifth finger (Table 6; Fig 7).

**Table 6:** Relative force differences for individual digits during the transportation of the three different sized posts (all weights combined, n = 192). Identified here are the proportion of resample cases more extreme than the difference recorded after 100,000 resamples without replacement (one tail). These values are analogous to a significant ‘*P*’ value, with significant differences (where cases more extreme than the observed difference are under 5% of the distribution) being highlight in bold. Positive values indicate the post detailed in the vertical column to have the greater of the comparative forces, while a negative value indicates the post in the horizontal row to be greater.

(n = 192)		Mean Force	
		Post 1	Post 2
**Thumb**	Post 2	-.0986	
	Post 3	**-.0001**	**-.0001**
**Index**	Post 2	-.2367	
	Post 3	**-.0004**	**-.0035**
**Middle**	Post 2	**-.0315**	
	Post 3	**-.0001**	**-.0001**
**Fourth**	Post 2	-.1945	
	Post 3	**-.0001**	**-.0001**
**Fifth**	Post 2	.2194	
	Post 3	.1710	-.0542

**Fig 8:**
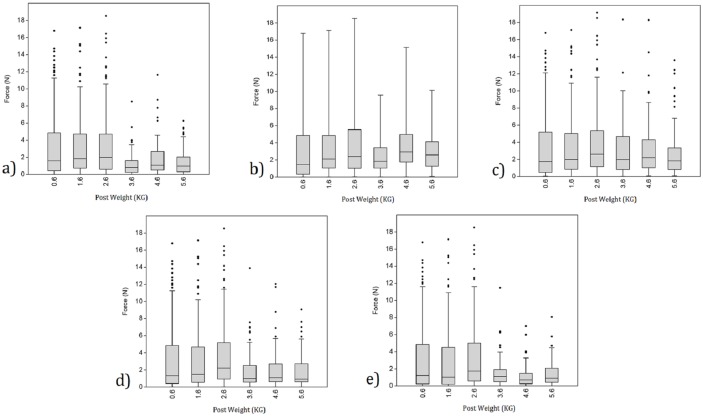
Boxplots identifying the mean force (N) recorded by individual digits during the transportation of the six post weights (all post sizes combined, n = 96). Images a, b, c, d, and e identify the thumb, index, middle, fourth and fifth fingers (respectively). It is worth noting that due to the scaling of the y axis on each figure any differences in the mean forces between weight categories appear to be more limited than they actually are.

For each digit there were fifteen possible force comparisons between posts of varying weight (i.e. the six different post weights compared against each other). Across all five digits, this resulted in a total of 75 resampling procedures. Of these, 49 exhibited a significant difference in a digit’s force record (Table 7). All but one of these identified the heaviest post in the comparison to have required the greater amount of manipulative force (Table 7). Weight comparisons that did not identify a significant difference were predominantly between posts that varied by 1-2kg and were more frequent as weights increased (Table 7). Indeed, forces tended not to increase when weight rose above 3.6kg (Table 3; Fig 8). In the case of the thumb there were a number of exceptions, as not only were there substantially fewer significant results returned (relative to the fingers), but in some instances, weight differences of up to 4kg did not elicit a significant difference in the amount of force recorded (Table 7; Fig 8).

**Table 7:** Relative force differences for individual digits during the transportation of the six different post weights (all sizes combined, n = 96). Identified here are the proportion of resample cases more extreme than the difference recorded after 100,000 resamples without replacement (one tail). These values are analogous to a significant ‘*P*’ value, with significant differences (where cases more extreme than the observed difference are under 5% of the distribution) being highlight in bold. Positive values indicate the weight detailed in the vertical column to have the greater of the comparative forces, while a negative value indicates the weight in the horizontal row to be greater.

	(n = 96)	Mean Force				
	Weight (kg)	0.6	1.6	2.6	3.6	4.6
Thumb	1.6	**-.0080**				
	2.6	-.1074	.1707			
	3.6	-.1130	.0921	.4168		
	4.6	**-.0002**	-.1286	**-.0236**	**-.0067**	
	5.6	**-.0052**	-.4875	-.1543	-.0764	**-.0064**
Index	1.6	**-.0001**				
	2.6	**-.0001**	-.4667			
	3.6	**-.0001**	**-.0061**	**-.0180**		
	4.6	**-.0001**	**-.0001**	**-.0001**	**-.0003**	
	5.6	**-.0001**	**-.0001**	**-.0005**	-.1172	**.0058**
Middle	1.6	**-.0030**				
	2.6	**-.0002**	-.1971			
	3.6	**-.0001**	**-.0008**	**-.0198**		
	4.6	**-.0001**	**-.0001**	**-.0029**	-.2312	
	5.6	**-.0001**	**-.0120**	-.1039	.2142	.0644
Fourth	1.6	-.0215				
	2.6	**-.0001**	**-.0150**			
	3.6	**-.0001**	**-.0006**	-.1526		
	4.6	**-.0001**	**-.0002**	-.1017	-.3991	
	5.6	**-.0001**	**-.0003**	-.1767	.4357	.3335
Fifth	1.6	.4797				
	2.6	**-.0002**	**-.0005**			
	3.6	**-.0001**	**-.0001**	**-.0229**		
	4.6	**-.0001**	**-.0001**	-.3423	.0521	
	5.6	**-.0001**	**-.0001**	**-.0133**	-.4678	**-.0346**

## Discussion

The manual transportation of objects (i.e. carrying) is a behavior widely observed amongst extant primates. Modern humans are, however, extreme in this regard, and transport objects over far greater distances than other primates. Certainly, humans may be considered exceptional in not only their ability to transport objects efficiently over long distances, but the relative importance placed upon this behavior within at least some populations [77–82]. Archaeological evidence suggests that the exceptional manifestation of this behavior is long lived within the hominin clade (e.g. [60, 62, 63]). It is, therefore, logical to ask whether the unique emphasise placed upon carrying behaviors by hominins [59, 64] played a role in the evolution of the unique suite of musculoskeletal adaptations observed in the human hand. To date, there has been a lack of information regarding any impact that this behavior may have had upon the evolutionary trajectory of the hominin hand. The present study sought to address this deficiency by examining force distribution in the hand during the manual transportation of objects relevant to the known behavioral repertoire of Plio-Pleistocene hominins.

The first set of analyses set out to test whether there were any significant differences in the forces experienced between digits during the transportation of four broad object classifications. Results identified a number of significant differences between digits, however, it was clear that the nature of any variation was dependent upon the object being carried (Table 5). Of the 22 significant between digit comparisons within the mean force data, 18 identify either the index or middle finger as being recruited to a greater extent (7 and 11 times each, respectively). Similarly, of the 22 significant differences exhibited within the maximum force data, 13 identify the index or middle finger to have recorded greater forces relative to other digits (4 and 9 times each, respectively). Hence, when examined at a broad level, the index and middle fingers had greater levels of force acting upon them than the other three digits in most instances. Musculoskeletal stresses associated with the manual transportation of materials in the Plio-Pleistocene are, then, likely to have focused upon the second and third digits (and notably not the first).

Force records, however, deviate between those returned for the round stones and those for the other three object classifications. Indeed, there are comparatively fewer significant differences for the former, and of those identified, half identify the index finger to have experienced lower maximum forces than the other digits (Table 5). This is surprising given the prominent role of the index finger during the transportation of the other object classifications. Further to the round stones, it is unusual that the thumb recorded significantly lower maximal forces during the transportation of the large two-handed stone relative to all other digits. Indeed, even when compared to the fifth digit, the thumb recorded far lower maximal forces when carrying this large and heavy object. The relative forces experienced through the thumb and fingers during carrying events can, then, clearly be dependent upon the type of object transported.

Different recrui^™^ent levels most likely result from the variable positioning of individual digits relative to each other, the palm, and the mass distribution of the object transported, which is in turn a likely consequence of differing object forms, weights and sizes [83–85]. Indeed, while participants typically utilized broad ‘power’ grips during most transportation events [47, 86], there was substantial variation in the form that these grips took and how they provided support to objects (Fig 2; S1 Fig). Fig 2F, for example, identifies the fingers as direct load bearers on the inferior surface of the large stone while the thumb has a more limited securing role on the superior surface (in turn helping to explain the differing maximal forces identified between the thumb and fingers for this object). Fig 2B and 2C, on the other hand, identify grips where the distal aspects of the fingers and thumb appear to have a similar role in securing an object with the hand. Video footage of the transportation events confirms there to be object-specific diversity in the types of grips applied by participants. For example, a review of the load-bearing role of the palm during carrying events reveals that the palm’s orientation varies dependent upon object type and weight/size (Table 8).

**Table 8:** Palm orientation during carrying events dependent upon the type of object transported.

		Palm Orientation	
		Down Turned (%)	Up Turned (%)
Round Stones (n = 32)	1	53	47
	2	38	62
	3	25	75
Flat Stones (n = 32)	1	50	50
	2	44	56
Large Stone (n = 32)	1	47	53
Posts (n = 192)	1	81	19
	2	73	27
	3	37	63

It is not immediately clear why such low maximal forces acted upon the index finger when individuals carried the round stones and yet displayed no significant differences in mean force. An explanation would likely be found if the grips used to pick up the stones in the first instance (where maximal forces tended to be recorded [Fig 4]) could be compared against those for the rest of the transportation event. Indeed, as Fig 4 demonstrates for the thumb, force contributions made by individual digits can alter throughout the duration of any transportation event (also see S1A and S1B Fig). While more detailed analyses of the relationships between manual forces and grip choices are not possible here, it is apparent that carrying behaviors have the potential to invoke substantial variation in utilized grips, and that this variation consequently affects distributions of manual force. Further, some of the grips displayed by participants are comparable to those observed during stone tool related activities (particularly cradle and power grips and cupping actions [S1 Fig]) and associated with the evolution of a number of anatomical features of the human hand [2, 11, 18, 22, 47]. More detailed investigation in the future of the grips employed during carrying activities may, then, shed light upon the evolution of these features (in particular the carpometacarpal region). Certainly, it is plausible that in addition to stone tool related behaviors [1], carrying may have contributed to the evolution of human gripping capabilities. The potential evolutionary significance of carrying is further highlighted by differences that appear to be present between the grips employed here and those utilized by chimpanzees during manual transportation events [87, 88]. For instance, although there are similarities such as the frequent use of ‘cup hold’ grips where the palm has a prominent supportive role and the digits form an inclusive cradle around the object (S1Q Fig), there are notable distinctions, including the direct and forceful opposition of the distal aspects of the thumb and fingers when an object is secured/suspended directly beneath the palm (e.g. S1G Fig). It is, however, important to reemphasise that more detailed comparative analyses of the grips employed by chimpanzees (and bonobos) and humans during carrying events are required in order to be able to comment further upon the potential evolutionary significance of any differences.

The second set of analyses examined the impact that variation in the size and weight of an object has upon the forces exerted and resisted by individual digits during carrying behaviors. Force data were collected from the wooden posts so that these two variables could be independently examined (i.e. object size could be examined while weight was controlled for, and vice versa). Results identified digits 1–4 to have recorded significantly more force during the transportation of the largest post relative to the two others with reduced diameters. Indeed, despite the maintenance of weight, increases in the diameter of the posts from 4.5 and 5.8cm up to 9.5cm resulted in significant increases in the force experienced by these four digits. Somewhat conversely, the relatively smaller increase in diameter from 4.5 to 5.8cm only resulted in increased force for the middle finger. On no occasion did increases to the size of a wooden post result in the fifth finger experiencing greater loading. It therefore appears that the digits most heavily recruited during carrying activities (i.e. digits 1–4 [Tables 4 and 7; Fig 6]) are those that are preferentially exposed to any additional stresses caused by increases to the size of an object. Indeed, the diminutive size of the fifth digit may preclude any necessary increases in force required by carrying larger objects. Similarly, the additional significant results returned for the third digit (between Post 1 and Post 2) may suggest that at times it is preferentially employed above all others (including the index finger) when securing objects in the hand during carrying events. A statement supported by the between digit force comparisons. This may, in part, be due to the greater length of the 3^rd^ digit. Post hoc examinations of the relative force recorded by individual digits dependent upon the size of round stone transported similarly identified both a relationship between increasing object size and greater forces (although there are, of course, weight increases as well), and a preferential emphasis being placed upon the recrui^™^ent of the middle finger (S2 Table).

Logically, increases to the weight of an object should result in greater levels of loading acting upon the thumb and fingers. Indeed, in order to maintain a secure grip upon an object, muscles should alter their force output at a rate appropriate to any variation observed in the object’s weight. It is not surprising, therefore, that increases to the weight of the wooden posts resulted in significant increases to the forces recorded by each digit. This is consistent with previous ergonomic research (e.g. [83, 89]). There is, however, a clear difference between the results returned for the thumb and those returned for the four fingers. Indeed, relative to the fingers, there were fewer significant differences for the thumb, with this including a number of relatively large variances in weight (up to 4kg). Hence, the greater manual forces associated with the transportation of heavier objects may preferentially be focused upon the fingers as opposed to the thumb. This is, at least in part, likely to be a result of the object carried in this analysis being a cylindrical post. Indeed, in many instances the fingers were positioned more inferiorly on the object than the thumb (e.g. Fig 2E) and therefore were preferentially likely to be exposed to variations in object weight. While further controlled investigations of object weight are required (i.e. when size is maintained), the post hoc examinations of the three round stones suggest the thumb to be recruited in a similar respect to the fingers (S2 Table).

Results further demonstrated that increases in weight were less likely to elicit significant differences in force when objects are heavier (i.e. a comparison between 0.6kg and 2.6kg may result in a significant difference while a comparison between 3.6kg and 5.6kg may not [Table 4; S2 Table]). This may be due to a shift in the type of grip applied to an object dependent upon its weight. Indeed, as weights increased, participants were more likely to shift the applied direction of their grip from the superior surface of the post (e.g. Fig 2B and 2E) to the inferior surface (e.g. Fig 2D), in turn increasing the weight bearing role of the palm and reducing the required force output by the digits (participants displayed an upturned palm 26% of the time when carrying the lightest posts (0.6 Kg) when compared to 50% of the time when carrying the heaviest posts (5.6 Kg) [n = 96]). Some individuals similarly altered their grip in response to increases in post size, whereby if the fingers were not long enough to secure the post within the hand, then the palm would be employed in a major weight-bearing role. In addition to previous publications (e.g. [1, 18, 47, 83, 84, 90]), the results presented here highlight the complex relationships that are present between an object’s form and weight, the grips applied to that object when securing it in the hand, and the musculoskeletal stresses experienced by individual digits.

### Carrying and the Evolution of the Hominin Hand

Modern humans are unique in the nature and extent of their manual transportation behaviors. Archaeological evidence demonstrates that the uniquely ‘human-like’ manifestation of this behavior (i.e. the transportation of objects over substantial distances) is long-lived within our evolutionary history, occurring at least as early as ~2.3 MYA ([63]). If carrying behaviors played a recurrent and important role in the survival strategies of early hominins, we may then expect to see the morphological manifestation of any associated selective pressures on hand anatomy from a relatively early point in our evolutionary history. Potentially predating the onset of lithic technology and coinciding with the evolution of habitual bipedalism (*c*.*f*. [82, 87]).

The potential advantages provided by an increased ability to manually transport objects has been discussed widely within literature relating to the evolution of bipedalism [82, 87, 91–94]. Much of this relates to the movement of food resources, with there being clear selective advantages to the effective and efficient transportation of food items over long distances, particularly when provisioning for offspring or to facilitate the consumption of food at a later date. Moreover, an ability to maximize the amount of food material able to be collected at any one time (i.e. the transportation of increased material volumes) would similarly be advantageous. As demonstrated within archaeological literature, raw material sources for stone tools are often sparse within landscapes and the energetic costs associated with the transportation of stone tools/stone materials can not only be substantial, but has been shown to influence both tool use and tool production behaviors (e.g. [95–99]). Hence, an ability to more efficiently transport stone materials (i.e. the optimization of transported loads relative to energy output) would likely have substantial benefits for Palaeolithic hominins. Similar arguments can be constructed relating to the transportation of other items of necessity (e.g. digging sticks, spears, bone tools); particularly within environments containing sparse organic resources. In sum, there is evidence to indicate that not only are hominins unique in the extent and nature of their carrying behaviors, but there may have been evolutionary advantages to individuals that were able to more efficiently and/or effectively undertake these behaviors.

Presented here is evidence to suggest that when carrying objects similar to those transported by Plio-Pleistocene hominins, manual forces are focused upon the index and middle finger. Any influence that carrying behaviors may have had upon the evolutionary trajectory of the human hand are, then, likely to have been focused on these two digits. In other words, if carrying did impact upon the evolution of the hominin hand, these two digits should display anatomical features associated with the exertion and resistance of manipulative forces. This is at odds with a number of aspects of modern human hand anatomy that identify the thumb as being most heavily adapted in this regard [2,4]. Indeed, the results presented here suggest that the musculoskeletal adaptations of the thumb associated with the exertion and resistance of high loading levels (relative to the fingers) are unlikely to be attributed to carrying behaviors. It is not that the thumb is not vital to successful carrying activities. Indeed, Fig 2 and S1 Fig demonstrate that it can a play an important role in securing objects within the hand; a factor aided by our distinctively long thumb relative to the length of our fingers [100]. Rather, the present results suggest that on a comparative level with the second and (in particular) third digits, the greater robusticity and muscular (force) potential of the thumb is unlikely to have been the result of manual transportation activities. Current models of hominin hand evolution that emphasise the role that stone tool production and use may have played in the evolution of our uniquely robust thumb anatomy do not, therefore, need to be altered in light the present results [1, 2, 17, 14, 18, 35]. Indeed, the present analysis reemphasises the important contribution that stone tool related behaviors likely had during the evolution of the robust human thumb.

Modern humans are, however, unique amongst extant primates in displaying a styloid process on the third metacarpal, a feature that prevents hyperextension of the third metacarpal and stabilizes the third metacarpophalangeal/carpo-metacarpal (CMC) joints ([101]). Previous research has suggested that the evolutionary origin of the styloid process may relate to the high forces experienced during stone tool production/use activities and throwing [2, 36, 101]. Given the prominent role of the third digit here (even when compared to the index finger), it would be reasonable to hypothesise that carrying may similarly have had a role in the evolution of the styloid process on the third metacarpal. Certainly, forces applied to the third digit while it is in a supportive ‘cradle’ grip (e.g. Fig 2A, 2D, 2F and 2E), for example, would attempt to dorsally rotate the third metacarpal around an axis at the CMC joint, something which is not easily possible with the presence of the styloid process [101]. The manual transportation of objects, particularly those of substantial weight or size that (as demonstrated here) lead to increased stress being placed upon the third metacarpal, could then be aided by the presence of a styloid process. Within Plio-Pleistocene contexts that necessitated the frequent or prolonged transportation of such materials, the presence of a styloid process may have provided an adaptive benefit to individuals. Current fossil evidence supports such a relationship as the earliest appearance of the third metacarpal styloid process is dated to 1.42 MYA and associated with *Homo erectus* (*s*.*l*.) [7], a species thought to display an increased emphasis upon long distance travel and transportation behaviors relative to earlier hominins [102–105]. It is, however, important to note that the present results are only suggestive of an adaptive relationship in this regard and further research examining the precise role of the 3^rd^ metacarpal styloid process during carrying behaviors is required. Further, it is worth noting that the relative requirement for the styloid process during the transportation of more manageable (i.e. smaller/lighter) objects is likely to be limited [101].

Recent analyses by Rolian et al. [17], Williams et al. [14] and Key and Dunmore [18] have examined the forces/pressures experienced by the hand during stone tool production. All identified the hand as being exposed to considerable musculoskeletal stress during this behavior. Indeed, pressures acting upon both the dominant and non-dominant hand can at times exceed 50N [14, 17, 18]. The results presented here similarly identify a manual behavior where hominins were at times likely to have been exposed to high manual forces. Importantly, these forces are highly dependent upon the object being transported and in the majority of cases would not have been greater than those experienced during stone tool production [14, 17, 18]. Certainly, comparisons between the mean maximal forces shown here and those recorded when gripping a hammer stone during flake detachments identify the latter to be substantially greater [14]. The relative influence of carrying behaviors upon the evolution of the human hand when viewed as part of the total manipulative repertoire of stone tool producing hominins could then be debatable.

As previously argued by Rolian et al. [17], however, there is a distinction to be made between the brief exposure to high manual forces resulting from hammer stone strikes, and the more consistent exposure experienced during other manual activities. On this basis, Rolian et al. [17] argue that the lower manual forces they recorded during ‘simulated’ flake tool use, relative to hammerstone strikes, may in fact have placed greater selective pressure upon hand morphology. Given the considerable distances that early hominins are known to have transported objects over (e.g. [63]), and the hours that this could have taken, it is reasonable to predict that few other activities could have created more prolonged manual forces for early hominins (including early flake tool use [106]). Hence, despite the more limited forces associated with carrying (relative to stone tool related behaviors), it is plausible that its associated manual stresses could have placed selective pressures upon hominin hand morphology. When combined with recent observations of extant apes undertaking forceful manual activities during nut-cracking and food consumption behaviours [37, 107], it appears that the production and use of stone tools would not necessarily have been novel in exposing hominins to manipulative forces or grips (not related to locomotion) that may have influenced the evolution of hand anatomy during the Plio-Pleistocene. Rather, any unique role that stone tool production/use may have had during the evolution of the hominin hand is likely to be down to how forces are distributed, the greater maximal forces experienced, and the diversity and distinctiveness of grips that were required.

## Supporting Information

S1 Fig Examples of the diversity of grips utilized during carrying events. Of specific note are images A and B that identify two distinct grips utilized by a participant during a single carrying event (Round Stone 3). Images B, C, D, E, F, G and H highlight the role of the thumb, identifying that in at least some instances this digit is likely to be resisting and exerting particularly high forces during manual transportation events. The substantial weight bearing role of the distal fingers (e.g. D, F, H, I, J, K) and palm (e.g. B, L, M, N, O, P, Q, R) is also evident.
(TIF)

S1 File Full details of the methods used to collect biometric data from participants. (DOCX)

S2 File Mean and maximum data values. (XLSX)

S1 Table Relative force differences between digits during the transportation of each post size (n = 192). Identified here are the proportion of resample cases more extreme than the observed difference value after 100,000 resamples without replacement (one tail). These values are analogous to a significant ‘P’ value, with significant differences (where cases more extreme than the observed difference are under 5% of the distribution) being highlight in bold. Positive values indicate the digit detailed in the vertical column to have the greater of the comparative forces, while a negative value indicates the digit in the horizontal row to be greater.
(DOCX)

S2 Table Relative force differences for individual digits during the transportation of the three round stones (n = 32). Identified here are the proportion of resample cases more extreme than the observed difference value after 100,000 resamples without replacement (one tail). These values are analogous to a significant ‘P’ value, with significant differences (where cases more extreme than the observed difference are under 5% of the distribution) being highlight in bold. Positive values indicate the stone detailed in the vertical column to have the greater of the comparative forces, while a negative value indicates the stone in the horizontal row to be greater.
(DOCX)
