# Supplementary figures and images for: Manual Loading Distribution During Carrying Behaviors: Implications for the Evolution of the Hominin Hand
Source: PLoS One. 2016 Oct 3;11(10):e0163801. doi: 10.1371/journal.pone.0163801 (PMC5047513; doi:10.1371/journal.pone.0163801)

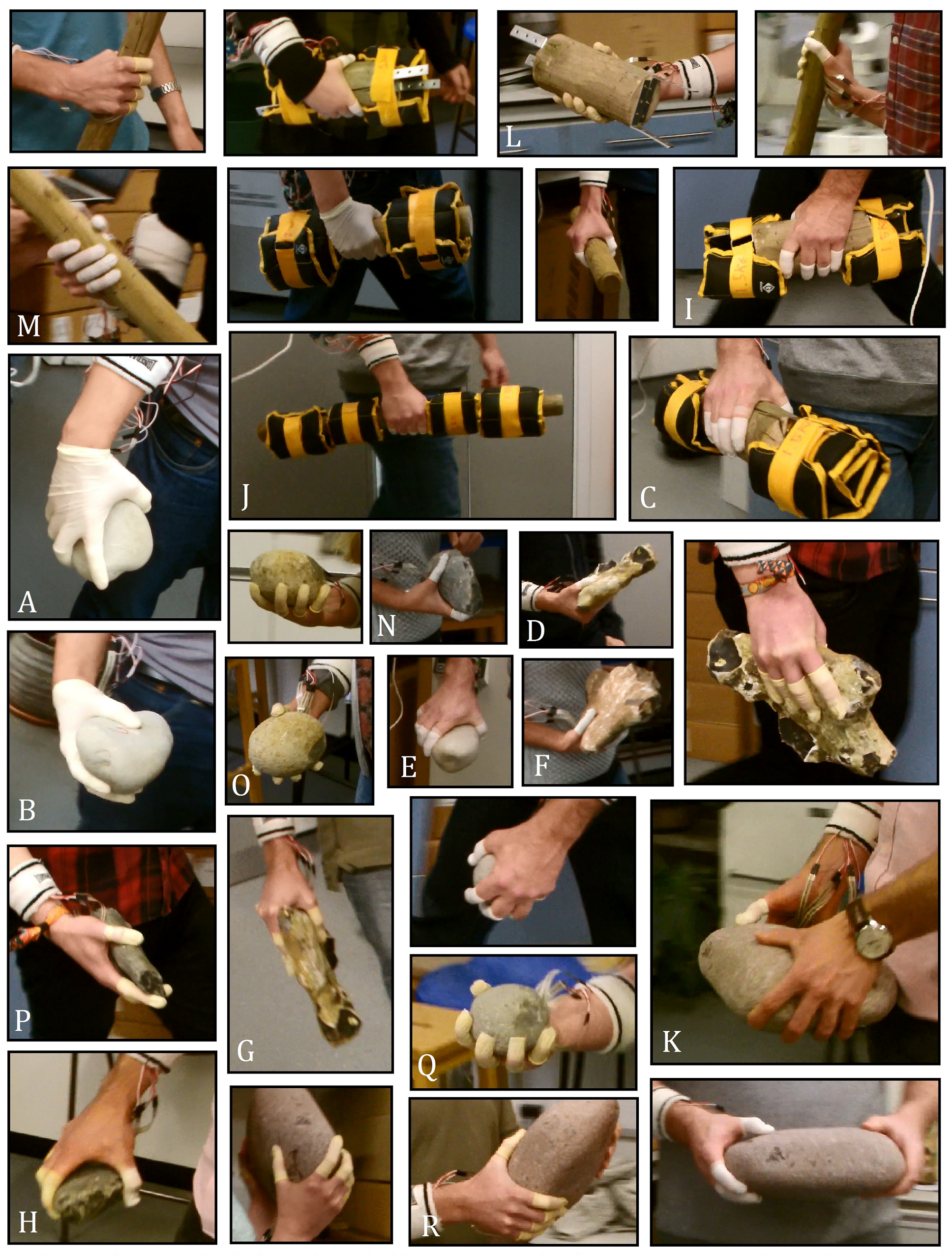

Supplement: S1 Fig — Of specific note are images A and B that identify two distinct grips utilized by a participant during a single carrying event (Round Stone 3). Images B, C, D, E, F, G and H highlight the role of the thumb, identifying that in at least some instances this digit is likely to be resisting and exerting particularly high forces during manual transportation events. The substantial weight bearing role of the distal fingers (e.g. D, F, H, I, J, K) and palm (e.g. B, L, M, N, O, P, Q, R) is also evident. (TIF) [file pone.0163801.s001.tif]
