# Supplementary material for: Manual Loading Distribution During Carrying Behaviors: Implications for the Evolution of the Hominin Hand
Source: PLoS One. 2016 Oct 3;11(10):e0163801. doi: 10.1371/journal.pone.0163801 (PMC5047513; doi:10.1371/journal.pone.0163801)
Supplement: S1 File — (DOCX) [file pone.0163801.s002.docx]

**Supplementary Information 1:** Details of the methods used to collect biometric data from participants.

‘Grip strength’ was recorded using a hand dynamometer in a transverse hook grip. ‘Pad-to-side’ pinch strength was recorded using a hydraulic pinch gauge, with the participant positioning the inferior surface of the ‘pinch point’ upon the joint of the proximal and intermediate second phalanges with the distal aspect of the thumb opposing the superior surface of the pinch point. ‘Tip-to-tip’ pinch strength was also recorded using the pinch gauge with the participants pinching the distal aspects of the first and second distal phalanxes, with the first digit opposing from the superior surface of the pinch point, while the second digit opposed from the inferior surface. The length of the thumb and index finger was recorded as the distance between their distal tip and the proximal palmer crease line at their intersection with the palm. ‘Hand length was recorded as the length of the supinated hand from the distal tip of the third digit to the first crease line at the wrist. Each variable was recorded prior to participants taking part in the experimental task.
