# Supplementary material for: Manual Loading Distribution During Carrying Behaviors: Implications for the Evolution of the Hominin Hand
Source: PLoS One. 2016 Oct 3;11(10):e0163801. doi: 10.1371/journal.pone.0163801 (PMC5047513; doi:10.1371/journal.pone.0163801)
Supplement: S1 Table — Identified here are the proportion of resample cases more extreme than the observed difference value after 100,000 resamples without replacement (one tail). These values are analogous to a significant ‘P’ value, with significant differences (where cases more extreme than the observed difference are under 5% of the distribution) being highlight in bold. Positive values indicate the digit detailed in the vertical column to have the greater of the comparative forces, while a negative value indicates the digit in the horizontal row to be greater. (DOCX) [file pone.0163801.s004.docx]

Supplementary Table 1: Relative force differences between digits during the transportation of each post size (n = 192). Identified here are the proportion of resample cases more extreme than the observed difference value after 100,000 resamples without replacement (one tail). These values are analogous to a significant ‘*P*’ value, with significant differences (where cases more extreme than the observed difference are under 5% of the distribution) being highlight in bold. Positive values indicate the digit detailed in the vertical column to have the greater of the comparative forces, while a negative value indicates the digit in the horizontal row to be greater.

|  | | Mean Force | | | | | | Maximum Force | | | | |
| --- | --- | --- | --- | --- | --- | --- | --- | --- | --- | --- | --- | --- |
| **Post Size** | **1** | (n = 192) | Thumb | Index | Middle | Fourth | Fifth | Thumb | Index | Middle | Fourth | Fifth |
|  |  | Index | **-.0001** |  |  |  |  | **-.0001** |  |  |  |  |
|  |  | Middle | **-.0001** | **.0002** |  |  |  | **-.0001** | .0569 |  |  |  |
|  |  | Fourth | -.1305 | **.0001** | **.0029** |  |  | **-.0039** | **.0001** | **.0098** |  |  |
|  |  | Fifth | -.0849 | **.0001** | **.0053** | -.4024 |  | **-.0001** | **.0251** | .3786 | .**0104** |  |
|  | **2** | (n = 192) | Thumb | Index | Middle | Fourth | Fifth | Thumb | Index | Middle | Fourth | Fifth |
|  |  | Index | **-.0001** |  |  |  |  | **-.0001** |  |  |  |  |
|  |  | Middle | **-.0001** | **.0049** |  |  |  | **-.0001** | .1185 |  |  |  |
|  |  | Fourth | -.2056 | **.0001** | **.0001** |  |  | **-.0580** | **.0001** | **.0004** |  |  |
|  |  | Fifth | .2460 | **.0001** | **.0001** | .1617 |  | **-.0283** | **.0001** | **.0021** | -.3491 |  |
|  | **3** | (n = 192) | Thumb | Index | Middle | Fourth | Fifth | Thumb | Index | Middle | Fourth | Fifth |
|  |  | Index | **-.0110** |  |  |  |  | .2830 |  |  |  |  |
|  |  | Middle | **-.0001** | **-.0002** |  |  |  | **-.0001** | **-.0001** |  |  |  |
|  |  | Fourth | -.1918 | .0915 | **.0001** |  |  | .2219 | .4365 | **.0001** |  |  |
|  |  | Fifth | **.0001** | **.0001** | **.0001** | **.0001** |  | **.0001** | **.0001** | **.0001** | **.0001** |  |
