# Supplementary material for: Manual Loading Distribution During Carrying Behaviors: Implications for the Evolution of the Hominin Hand
Source: PLoS One. 2016 Oct 3;11(10):e0163801. doi: 10.1371/journal.pone.0163801 (PMC5047513; doi:10.1371/journal.pone.0163801)
Supplement: S2 Table — Identified here are the proportion of resample cases more extreme than the observed difference value after 100,000 resamples without replacement (one tail). These values are analogous to a significant ‘P’ value, with significant differences (where cases more extreme than the observed difference are under 5% of the distribution) being highlight in bold. Positive values indicate the stone detailed in the vertical column to have the greater of the comparative forces, while a negative value indicates the stone in the horizontal row to be greater. (DOCX) [file pone.0163801.s005.docx]

Supplementary Table 2: Relative force differences for individual digits during the transportation of the three round stones (n = 32). Identified here are the proportion of resample cases more extreme than the observed difference value after 100,000 resamples without replacement (one tail). These values are analogous to a significant ‘*P*’ value, with significant differences (where cases more extreme than the observed difference are under 5% of the distribution) being highlight in bold. Positive values indicate the stone detailed in the vertical column to have the greater of the comparative forces, while a negative value indicates the stone in the horizontal row to be greater.

| (n = 32) | | **Mean Force** | |
| --- | --- | --- | --- |
|  |  | Round Stone 1 | Round Stone 2 |
| **Thumb** | Round Stone 2 | **-.0001** |  |
|  | Round Stone 3 | **-.0006** | .1469 |
| **Index** | Round Stone 2 | **-.0168** |  |
|  | Round Stone 3 | **-.0006** | -.0668 |
| **Middle** | Round Stone 2 | **-.0001** |  |
|  | Round Stone 3 | **-.0001** | **-.0005** |
| **Fourth** | Round Stone 2 | **-.0001** |  |
|  | Round Stone 3 | **-.0001** | -.2925 |
| **Fifth** | Round Stone 2 | **-.0042** |  |
|  | Round Stone 3 | **-.0001** | -.0501 |
